# Enriched Pyridinic Nitrogen Atoms at Nanoholes of Carbon Nanohorns for Efficient Oxygen Reduction

**DOI:** 10.1038/s41598-019-56770-8

**Published:** 2019-12-27

**Authors:** Jae-Hyung Wee, Chang Hyo Kim, Hun-Su Lee, Go Bong Choi, Doo-Won Kim, Cheol-Min Yang, Yoong Ahm Kim

**Affiliations:** 1 Institute of Advanced Composite Materials, Korea Institute of Science and Technology (KIST), 92 Chudong-ro, Bongdong-eup, Wanju-gun, Jeollabuk-do 55324 Republic of Korea; 2 Alan G. MacDiarmid Energy Research Institute, Department of Polymer Engineering, Graduated School & School of Polymer Science and Engineering, Chonnam National University, 77 Yongbong-ro, Buk-gu, Gwangju 61186 Republic of Korea

**Keywords:** Energy science and technology, Materials science, Nanoscience and technology

## Abstract

Nitrogen (N)-doped nanostructured carbons have been actively examined as promising alternatives for precious-metal catalysts in various electrochemical energy generation systems. Herein, an effective approach for synthesizing N-doped single-walled carbon nanohorns (SWNHs) with highly electrocatalytic active sites via controlled oxidation followed by N_2_ plasma is presented. Nanosized holes were created on the conical tips and sidewalls of SWNHs under mild oxidation, and subsequently, the edges of the holes were easily decorated with N atoms. The N atoms were present preferentially in a pyridinic configuration along the edges of the nanosized holes without significant structural change of the SWNHs. The enriched edges decorated with the pyridinic-N atoms at the atomic scale increased the number of active sites for the oxygen reduction reaction, and the inherent spherical three-dimensional feature of the SWNHs provided good electrical conductivity and excellent mass transport. We demonstrated an effective method for promoting the electrocatalytic active sites within N-doped SWNHs by combining defect engineering with the preferential formation of N atoms having a specific configuration.

## Introduction

Single-walled carbon nanohorns (SWNHs) are *sp*^2^-bonded carbon allotropes, similar to short single-walled carbon nanotubes (SWNTs). An individual SWNH has a closed tubular structure 2–5 nm in diameter and 40–50 nm in length, and SWNHs aggregate to form a spherical cluster 80–100 nm in diameter^1^. The conical edges and sidewalls of SWNHs exhibit high chemical reactivity owing to the presence of pentagonal and hexagonal defects, which induce the pyramidal distortion of *sp*^2^ carbon bonds^2–5^. Furthermore, SWNHs can have high porosity by forming hole defects that facilitate the entry of molecules or ions to the confined tubular inner space via partial oxidation^6–9^. Consequently, the physical and chemical properties of carbon materials such as the electrical conductivity, chemical activity, and porosity can be tailored by selective introduction of chemical moieties and defects on the conical edges and sidewalls of SWNHs.

Nitrogen (N)-doped carbon nanomaterials have been actively evaluated as promising alternatives for precious-metal catalysts in various electrochemical energy generation systems^10^. Recently, N-doped SWNHs have been proposed as exciting materials in an electrochemical capacitor^11^, a catalyst metal-supporting substance for a fuel cell^12,13^ and a catalyst in the oxygen reduction reaction (ORR)^14,15^.

Thus far, among the numerous ways of introducing N atoms into carbon nanomaterials, three methods have been reported for synthesizing N-doped SWNHs: using an arc discharge with a carbon and nitrogen source^11,14,16,17^, laser ablation of graphite in a N_2_ atmosphere^18^, and thermal treatment with a N-containing source^13,15^. Unni *et al*. synthesized N-doped SWNHs by thermally treating hydrogen peroxide-functionalized SWNHs with urea^15^. In this case, the N doping efficiency was significantly limited owing to the perfect *sp*^2^-structure, and the doping process increased the specific surface area of the SWNHs. Evaluating the altered properties via N doping is complicated because of the complex composition of doped N and the structural changes that accompany the doping process. Even though the selective regulation of the N configuration on the surface of SWNHs is essential for controlling the surface chemistry, it is difficult to achieve using the previously reported methods. Therefore, the synthesis of SWNHs having a specific N configuration while maintaining the initial pore structure is highly important for an in-depth study of the electrocatalytic active sites of N-doped SWNHs.

Herein, we propose an effective method for synthesizing N-doped SWNHs with highly electrocatalytic active sites via controlled oxidation followed by N_2_ plasma treatment. Under mild oxidation, nanosized holes were generated on the sidewalls and tips of SWNHs, and subsequently, the edges of the holes were easily decorated with N atoms. The N-doped defective SWNHs with pyridine rich configurations exhibited significantly improved electrocatalytic performance while retaining their initial structure.

## Results and Discussion

**Figure 1:**
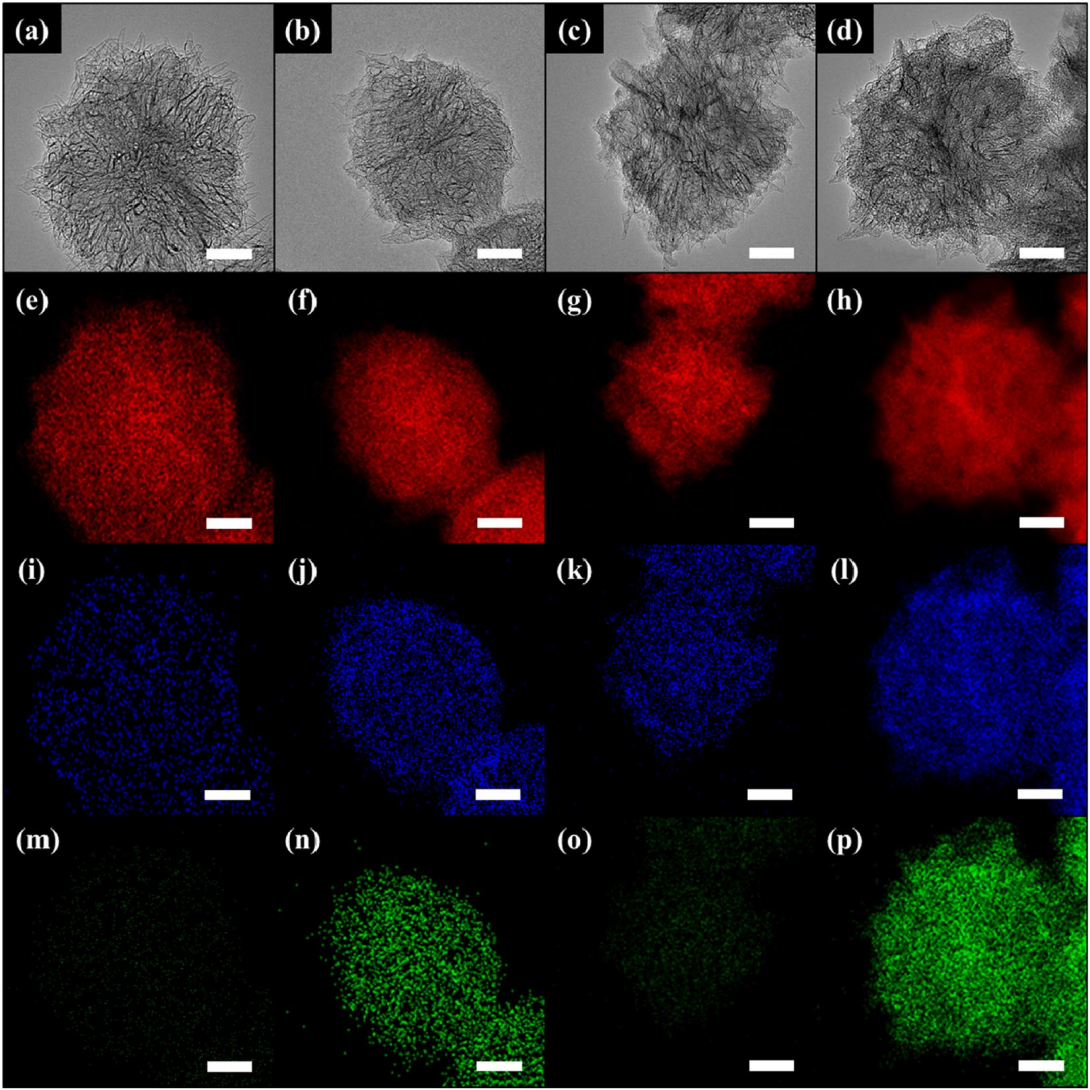
HRTEM images of (**a**) SWNHs, (**b**) N-SWNHs, (**c**) O-SWNHs, and (**d**) N-O-SWNHs and their corresponding STEM-EDX maps (**e**–**p**). In the STEM-EDX maps, red, green, and blue correspond to C (**e–h**), O (**i–l**), and N (**m–p**), respectively, and the scale bar represents 20 nm.

The morphological change of the SWNHs after the post-treatments (oxidation and N_2_ plasma) was observed using HRTEM. As shown in Fig. 1(a), the pristine SWNH bundle had a dahlia-like structure with a diameter of 80 nm and there is no metallic impurities^1^. The shape of the individual SWNHs was similar to that of an SWNHs with a closed tip (Fig. S1(a)). Even though there was no discernable change in the bundle structure after oxidation (Fig. 1(c)), it was previously reported that hole-like defects were created on the sidewalls and tips of individual SWNHs (Fig. S1(b))^9^. Additionally, there was no noticeable change in the bundle structure when the pristine and oxidized SWNTs were exposed to N_2_ plasma for 30 min (Fig. 1(b,d)). The elemental distribution within the SWNHs was verified using STEM-EDX. The SWNHs and O-SWNHs were mainly composed of carbon (C) and oxygen (O) (Fig. 1(e,i,g,k)), whereas N was identified in the N_2_ plasma-treated samples (Fig. 1(n,p)). Notably, the N was widely distributed within the N-O-SWNHs compared with the N-SWNHs.

**Figure 2:**
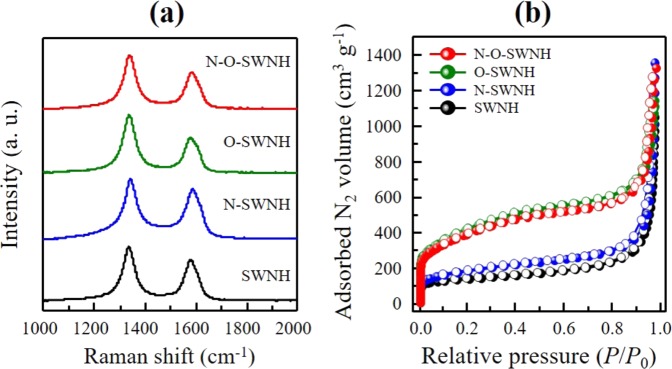
(**a**) Raman spectra obtained using a 514 nm laser line and (**b**) N_2_ adsorption isotherms at 77 K for the pristine and N-doped SWNHs (closed symbol: adsorption, open symbol: desorption).

Raman spectroscopy is a useful technique for evaluating the electronic properties and structural integrity of carbon materials^19,20^. The Raman spectra for SWNHs before and after the post-treatments were measured using a 514 nm laser line (Fig. 2(a)). All the samples exhibited two major peaks at 1336 cm^−1^ (D-band) and 1578 cm^−1^ (G-band). To evaluate the effects of the post-treatments on the defect concentration (or structural integrity) of the SWNHs (Table 1), the *R* value (*I*_D_/*I*_G_, i.e., the integrated intensity of the D-band divided by that of the G-band) was calculated. The *R* value of the pristine sample was found to be 1.34. This high *R* value is explained by the characteristic dahlia-like structure of the SWNH, which contained pentagonal and heptagon defects^21^. After oxidation on SWCNHs, the *R* value of O-SWNHs increases from 1.34 to 1.66 owing to the creation of hole defects, which is attributed to the increased density of defect sites on the conical edges and sidewalls of SWNHs due to oxidation by nitronium ions. N_2_ plasma treatment induced a slight decrease in the *R* value, possibly due to the thermal effect. However, N-doping on SWNHs caused structural deformations, such as wrinkles and less crystalline forms, resulting in higher concentrations of defects than the initial state^7,8,22^. The frequencies of the D- and G-bands shifted to higher levels (by approximately 4 and 9 cm^−1^, respectively) upon N doping, which is indirect evidence for electron-acceptor doping^21,23–25^. Thus, the N atoms in the nanohorns acted as electron acceptors.

**Table 1:** Pore structure parameters and *R* values obtained via Raman spectroscopy for the pristine and N-doped SWNHs.

	Pore structure parameters (SPE method)						Raman
	S_total_^[a]^ (m^2^ g^−1^)	S_mic_^[b]^ (m^2^ g^−1^)	S_ext_^[c]^ (m^2^ g^−1^)	V_total_^[d]^ (mL g^−1^)	V_mic_^[e]^ (mL g^−1^)	V_ext_^[f]^ (mL g^−1^)	*R* value^[g]^
SWNH	425	255	170	1.06	0.13	0.92	1.34
N-SWNH	509	263	246	1.19	0.17	1.02	1.19
O-SWNH	1197	818	379	1.50	0.54	0.96	1.66
N-O-SWNH	1082	721	360	1.72	0.50	1.22	1.48

*^[a]^S_total_: total specific surface area, ^[b]^S_mic_: micropore surface area, ^[c]^S_ext_: external surface area, ^[d]^V_total_: total pore volume, ^[e]^V_mic_: micropore volume, ^[f]^V_ext_: external pore volume, ^[g]^*R* value: integrated-intensity ratio of the D-band to the G-band in Raman spectra.

To evaluate the change in the porosity of the SWNHs after the post-treatments, we measured N_2_ adsorption isotherms at 77 K (Fig. 2(b)). The N_2_ adsorption isotherms of the SWNHs were found to be Type-II, which are representative adsorption isotherms on flat surfaces. It is well known that tube-shaped cylindrical micropores in our sample do not exhibit hysteresis loops^6,26,27^. Even though the oxidation did not change the type of the N_2_ adsorption isotherm, we observed a significantly increased amount of N_2_ adsorbed in the O-SWNHs at a lower relative pressure than the pristine sample. Additionally, the N_2_ plasma treatment did not significantly change the adsorption isotherms. To evaluate the pore structure in detail, we obtained the porosity parameters via the SPE method using α_s_-plots obtained from the N_2_ adsorption isotherms (Table 1)^28^. The pristine SWNHs exhibited a completely closed tip structure; thus, N_2_ molecules could only be adsorbed in the interstitial spaces between adjacent SWNHs and on the external surfaces of individual SWNHs. However, the oxidized sample exhibited significant increases in both the total surface area (1197 m^2^ g^−1^) and the micropore volume (0.54 mL g^−1^), owing to the hole opening on the conical tips and sidewalls of the SWNHs due to the controlled oxidation^8^. The development of the internal porosity for the O-SWNHs is supported by the increase of the R value in the Raman spectra and the hole defects in the HRTEM images. There was no significant (>10%) change in the porosity parameters including micropore size distribution after the N_2_ plasma treatment^29,30^, indicating that the structural integrity was preserved during the N_2_ plasma doping process.

**Figure 3:**
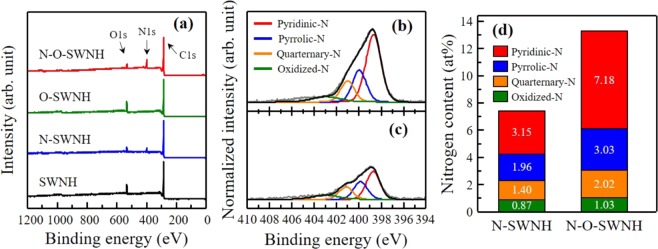
(**a**) Wide-scan XPS for the pristine and N-doped SWNHs, deconvoluted N 1s spectra for (**b**) N-O-SWNHs and (**c**) N-SWNHs, and (**d**) N component distribution obtained using the deconvoluted peak area ratio.

To investigate the amount of foreign atoms and their bonding configurations after the post-treatments, we performed XPS (Fig. 3) and examined the elemental compositions before and after the N_2_ plasma treatment (Table S1). All the spectra were normalized according to the C 1s spectra maximum peak (Fig. S2(a)). The N 1s peak for the N_2_ plasma-treated samples indicates the successful incorporation of N atoms into the SWNHs (Fig. 3(a)). The amounts of N atoms for the N-SWNHs and N-O-SWNHs were found to be 7.32 and 13.26 at%, respectively. To identify the configuration of the N atoms, the N 1s spectra for the N_2_ plasma-treated sample were deconvoluted into four peaks (Fig. 3(b,c)). The four peaks at 398.5, 399.8, 401.2, and 403.2 eV can be assigned to pyridinic-N, pyrrolic-N, quaternary-N, and oxidized-N, respectively^13,31,32^. Interestingly, the pyridinic-N content of the N-O-SWNHs was predominant (7.18 at%, which is more than twice that of the N-SWNHs) (Fig. 3(d)). These results are explained by the defect creation on both conical edges and sidewalls (see Raman spectra in Fig. 2(a)), as well as the substantial increase in the O content, including O-functional groups (Fig. S2(b)), because of the controlled oxidation. Gong *et al*. demonstrated that imperfections of carbon structures, such as defects, edges, and functionalized carbon atoms, are energetically beneficial for the introduction of N atoms compared with the basal plane on the surface of carbon materials^33^. In our case, the selective introduction of N moieties into the N-SWNHs can be explained by the high chemical reactivity of pentagonal and heptagonal defects, which are commonly observed in the conical edges and sidewalls of SWNHs^4^. Additionally, the substantial enrichment of the pyridinic-N for the N-O-SWNHs is explained by the fact that pyridinic-N is energetically more favorable than other N configurations because the reaction free energy of the defective sites is low with regard to the N atoms^34–36^. After the N-doping, the C 1s peak positions for the N-SWNHs and N-O-SWNHs were slightly downshifted to approximately 284.5 and 284.4 eV, respectively (Fig. S2(a)), which is a characteristic of doped materials having a p-type band structure, e.g., pyridinic-N-doped CNTs and graphene^25,37^. As seen in Fig. S4, no significant crystallographic structure change was also confirmed in XRD. Hence, our results indicate that the pyridinic-N atoms were predominantly introduced into the edges of defects (holes) without causing any significant structural change.

**Figure 4:**
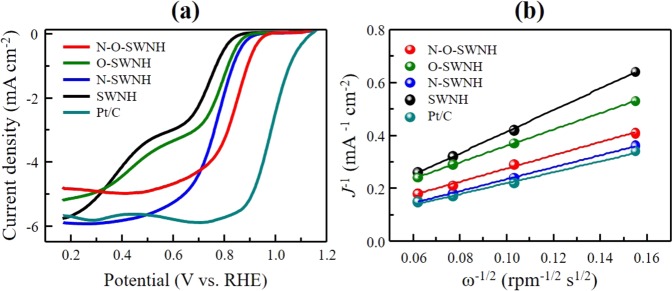
(**a**) Linear sweep voltammograms obtained in 0.1 M O_2_-saturated KOH aqueous electrolyte at a rotation speed of 1600 rpm (scan rate of 5 mV s^−1^) and (**b**) K -L plots of the pristine and N-doped SWNHs obtained at a potential of 0.6 V vs. RHE.

Finally, to evaluate the role of the pyridinic-N atoms as electrocatalytic active sites, we measured the LSV curves for the pristine and N-doped samples in an O_2_-saturated 0.1 M KOH aqueous electrolyte (Pt/C (20 wt%) was used for reference). Figure 4(a) shows the LSV curves for all the samples, which were obtained using a rotation speed of 1600 rpm at sweep rates of 5 mV s^−1^. The LSV curve for the pristine sample shows two limiting current densities, which is a typical LSV curve shape for a reaction with two onset potentials, as previously reported for CNTs and graphene materials^15,38^. In contrast, the N-doped samples exhibited only one limiting current density, similar to the reference Pt/C sample. The onset potential of the O-SWNHs was 0.84 V (that of the pristine SWNHs was 0.82 V), indicating the increase in the ORR active sites due to the increase in the specific surface area^39^. After the N-doping, the onset potential of the N-SWNHs and N-O-SWNHs were 0.86 and 0.91 V, respectively. The positive shift in the onset potential for the N-doped samples indicates the enhancement in their intrinsic ORR activity without the reduction of the limiting current density. Interestingly, the N-O-SWNHs had a more positive onset potential than the N-SWNHs, although their limiting current density was lower. To elucidate the ORR mechanism of the pristine and N-doped samples, we constructed K–L plots (Fig. 4(b)) from LSV curves obtained using various rotation speeds of the electrode in the range of 400–2500 rpm (Fig. S3). The slopes of the K–L plots for the N_2_ plasma-treated samples were lower than those for the pristine and oxidized samples; furthermore, they were very close to that for Pt/C. This clearly indicates that the electron-transfer pathway was shifted from two-electron (2e^−^) to four-electron (4e^−^) transfer kinetics via the N_2_ plasma treatment. The electron transfer numbers for the N-SWNHs and N-O-SWNHs were calculated to be 4.06 and 3.49 using the slopes of the K–L plots at 0.6 V vs. RHE. This significant improvement in the ORR performance of the N-doped samples indicates that the pyridinic-N atoms that easily and preferentially introduced via defect engineering promoted the oxygen reduction process. It was recently reported that pyridinic-N atoms transform the 2e^−^ pathway into a 4e^−^ reduction pathway and that quaternary-N can increase the limiting current^40–42^. Our results indicate that the creation of holes (defects) on the conical edges and sidewalls of the SWNHs led to the preferential formation of pyridinic-N, significantly enhancing both the onset potential and the electron-transport capability by retaining electrocatalytically active sites. However, a small amount of quaternary-N relative to pyridinic-N lowers limiting current density. Therefore, the judicious combination of defect engineering with balanced N configurations (i.e., pyridinic- and quaternary-N) should be employed to clarify the effects of the N configuration on the electrocatalytic active sites of N-doped nanostructured carbons.

## Conclusion

We synthesized N-doped SWNHs via controlled defect creation on the conical edges and sidewalls under mild oxidation, followed by N_2_ plasma treatment. The N-doped SWNHs retained their initial morphology, without significant structural destruction. The N introduced in the SWNHs was predominantly pyridinic-N, and the oxidized (defective) SWNHs exhibited a pyridinic-N content twice as high as that of the pristine SWNHs. The ORR performance was improved by N doping, indicating that the pyridinic-N atoms introduced via defect engineering acted as electrocatalytic active sites. A high content of pyridinic-N has been shown to induce ohmic losses by increasing the amount of scattering sites. The enriched edges decorated with pyridinic-N at the atomic scale increased the number of active sites for the ORR while the inherent spherical three-dimensional feature of the SWNHs provided good electrical conductivity and excellent mass transport. We demonstrated an effective method for increasing the electrocatalytic active sites in N-doped SWNHs through defect engineering via N doping with a specific configuration.

## Methods

### Preparation of materials

The as-grown SWNHs were synthesized via CO_2_ laser ablation of a graphite target in an Ar atmosphere. Oxidized SWNHs (O-SWNHs) were prepared by immersing 100 mg of SWNHs in 100 mL of 69% nitric acid and stirring for 48 h at room temperature. The precipitate was filtered and then washed three times with distilled water. Then, the collected sample was dried in vacuum for 12 h at 393 K. To introduce the N atoms into the carbon structure, SWNHs and O-SWNHs were exposed to the inductively coupled N_2_ plasma for 30 min. The N_2_ plasma was generated by applying a radiofrequency of 13.56 MHz with a power of 100 W under a 10 sccm flow of N_2_ gas at a pressure of 10 mTorr through a quartz tube with a diameter of 50 mm. The plasma-treated SWNHs and O-SWNHs were denoted as N-SWNHs and N-O-SWNHs, respectively.

### Structural characterization

To confirm the structural changes of the SWNHs after the oxidation treatment and N_2_ plasma treatment, high-resolution transmission electron microscopy (HRTEM, Titan^3^ G2 operated with 80 kV) was performed. Energy-dispersive X-ray spectroscopy (EDX) mapping was performed in the high-angle annular dark-field scanning transmission electron microscopy (STEM) mode at 80 kV using a Super-X 4-SDD system. Raman spectroscopy (Renishaw Invia, 514 nm laser) was performed to evaluate the electronic properties and structural integrity of the pristine and N-doped SWNHs. The pore structure changes of the samples were investigated via N_2_ adsorption measurements at 77 K using volumetric equipment (BELSROP-max, MicrotracBEL) after pre-evacuation for 6 h at 393 K, while the pressure was maintained at 10^–2^ Pa. The pore structure parameters were obtained via the subtracting pore effect (SPE) method. The SPE method was performed using high-resolution αs-plots comprising standard adsorption data for nonporous carbon black (Mitsubishi 4040 B). X-ray diffraction analysis (XRD, Rigaku operated with 200 mA at 45 kV) was performed to observe the overall changes in crystallinity. X-ray source was used Cu Kα. To evaluate the chemical configuration of the N-doped samples, X-ray photoelectron spectroscopy (XPS, K-Alpha, Thermo Fisher Scientific) was performed. The N 1s XPS spectra were deconvoluted by fitting the peak maximum within +0.2 eV and applying a full width at half maximum of 1.50 eV. The fitting value of the mixed Gaussian–Lorentzian was fixed at 30%.

### Electrochemical measurements

Electrochemical measurements were performed on the samples with a standard three-electrode system using an Autolab potentiostat/galvanostat. A Ag/AgCl electrode in 3.0 M KCl and Pt wire were used as the reference electrode and counter electrode, respectively. To prepare the working electrode, 10 mg of samples was added to the solution mixture of 1 mL of isopropyl alcohol and 100 μL of Nafion (5% w/w), followed by sonication for 30 min at 25 °C. Then, 10 μL of the obtained slurry was dropped onto a glassy carbon electrode and dried at room temperature. The ORR properties were evaluated via linear sweep voltammetry (LSV) in an O_2_-saturated 0.1 M KOH aqueous electrolyte using a rotating disk electrode (RDE). The voltage sweep rate was 5 mV s^−1^. We also carried out cyclic voltammetry and impedance analysis in N_2_ bubbled 0.1 M KOH electrolyte before ORR test for eliminating capacitive effects and iR compensated. The potential was transferred to reversible hydrogen electrode (RHE) via following equation;

1 $${E}_{RHE}={E}_{Ag/AgCl}+0.1976+0.059\times pH$$

The electron transfer number per O_2_ molecule was obtained using the Koutecký–Levich (K–L) equation^15,43^:

2 $${J}^{-1}=B{\omega }^{-0.5}+{{J}_{K}}^{-1}$$

where *J* and *ω* represent the obtained current density and the RDE rotational rate, respectively. The *J*_K_^−1^ and *B* values were obtained from a K–L plot, by plotting a straight line around the *J*^−1^ and *ω*^−0.5^ axes of this equation. Once *B* was determined, the electron number (*n*) was calculated using the Levich equation:

3 $$n=B/0.62F{({D}_{{O}_{2}})}^{2/3}{v}^{-1/6}{C}_{{O}_{2}}$$

where *F* is the Faraday constant (96485 C mol^−1^), ${D}_{{O}_{2}}$ is the O_2_ diffusion coefficient (1.9 × 10^−5^ cm^2^ s^−1^) in the 0.1 M KOH aqueous electrolyte, *v* is the kinetic viscosity (0.01 cm^2^ s^−1^), ${C}_{{O}_{2}}$ is the O_2_ concentration (1.2 × 10^−6^ mol cm^−3^) in the electrolyte, and 0.62 is a constant applied for expressing the rotational speed in revolutions per minute (rpm).

## Supplementary Information

Supplementary Information.
