# Supplementary material for: Enriched Pyridinic Nitrogen Atoms at Nanoholes of Carbon Nanohorns for Efficient Oxygen Reduction
Source: Sci Rep. 2019 Dec 27;9:20170. doi: 10.1038/s41598-019-56770-8 (PMC6934446; doi:10.1038/s41598-019-56770-8)
Supplement: Supplementary file 1 — Supplementary Information. [file 41598_2019_56770_MOESM1_ESM.doc]

**Supplementary information for**

Enriched Pyridinic Nitrogen Atoms at Nanoholes of Carbon Nanohorns for Efficient Oxygen Reduction

Jae-Hyung Wee^1,2^, Chang Hyo Kim^1^, Hun-Su Lee^1^, Go Bong Choi^2^, Doo-Won Kim^1^, Cheol-Min Yang^1,*^, Yoong Ahm Kim2,*[[1]](#footnote-2)

^1^Institute of Advanced Composite Materials, Korea Institute of Science and Technology (KIST), 92 Chudong-ro, Bongdong-eup, Wanju-gun, Jeollabuk-do, 55324, Republic of Korea

^2^Alan G. MacDiarmid Energy Research Institute, Department of Polymer Engineering, Graduated School & School of Polymer Science and Engineering, Chonnam National University, 77 Yongbong-ro, Buk-gu, Gwangju, 61186, Korea.


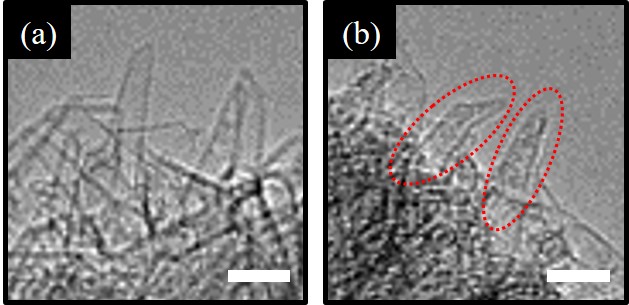


Figure S1. Individual nanohorn HRTEM images of (a) SWNHs and (b) O-SWNHs. Scale bar is 5 nm.

Figure S2. (a) C1s spectra and (b) O 1s spectra of the pristine and nitrogen-doped carbon nanohorns.


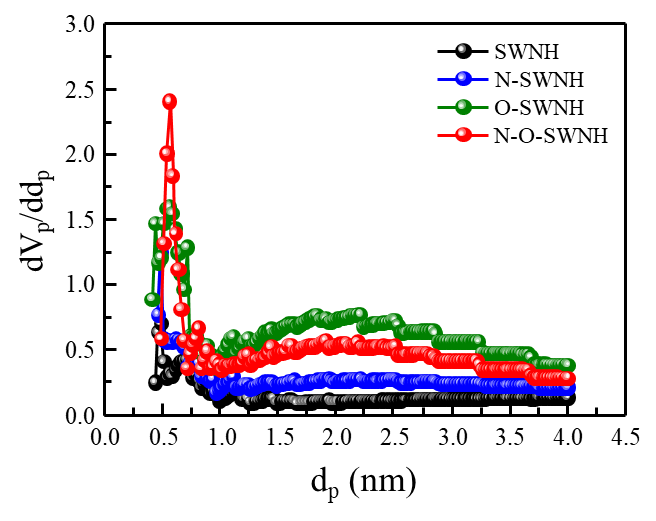


Figure S3. Micropore size distribution of SWNH, N-SWNH, O-SWNH, and N-O-SWNH by HK-plot.


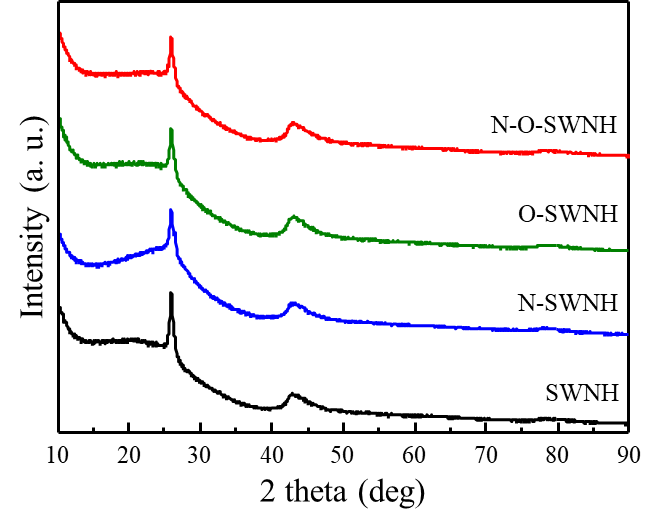


Figure S4. X-ray diffraction patterns of SWNH, N-SWNH, O-SWNH, and N-O-SWNH, respectively.


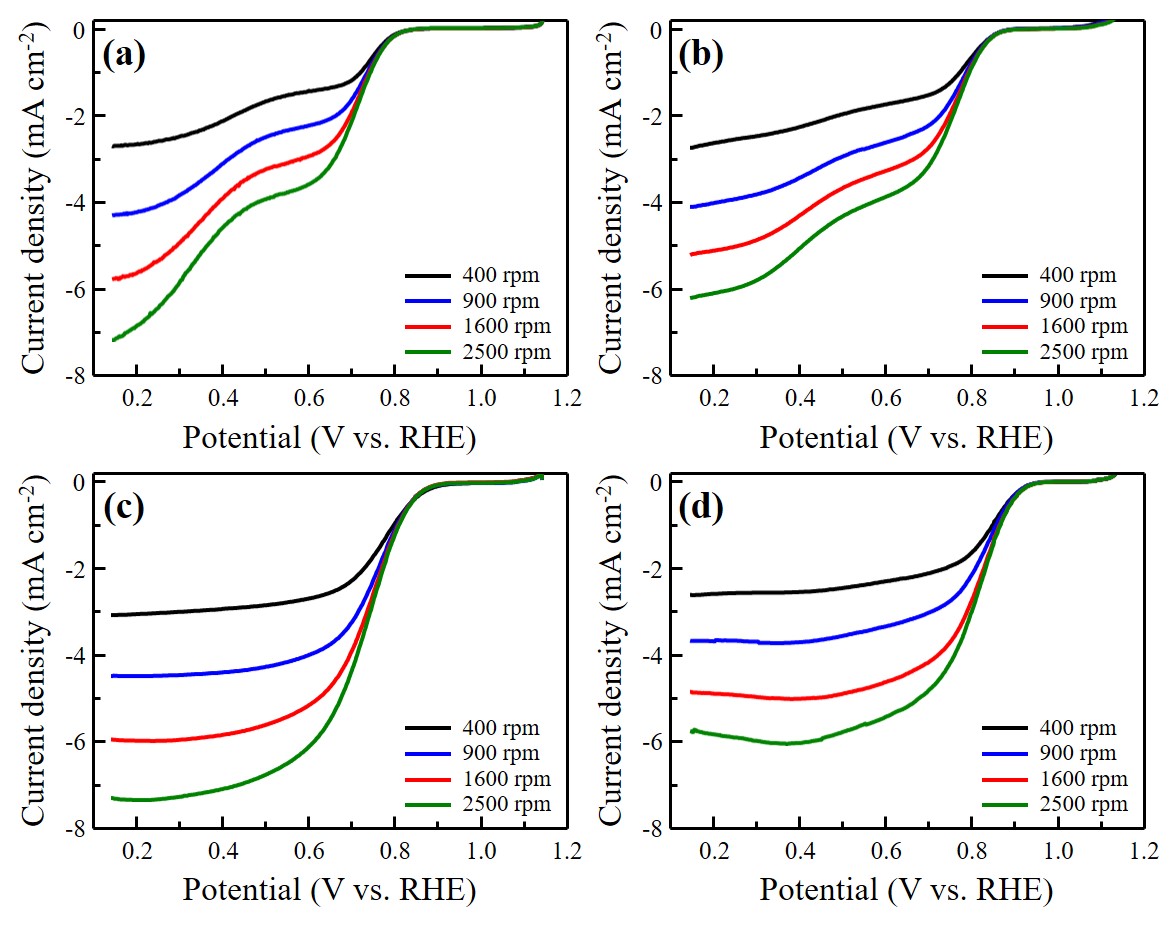


Figure S5. Linear sweep voltammograms of (a) SWNH, (b) O-SWNH, (c) N-SWNH, and d) N-O-SWNHs in 0.1 M O_2_-saturated KOH at various rotation speed (at scan rate of 5 mV s^-1^).


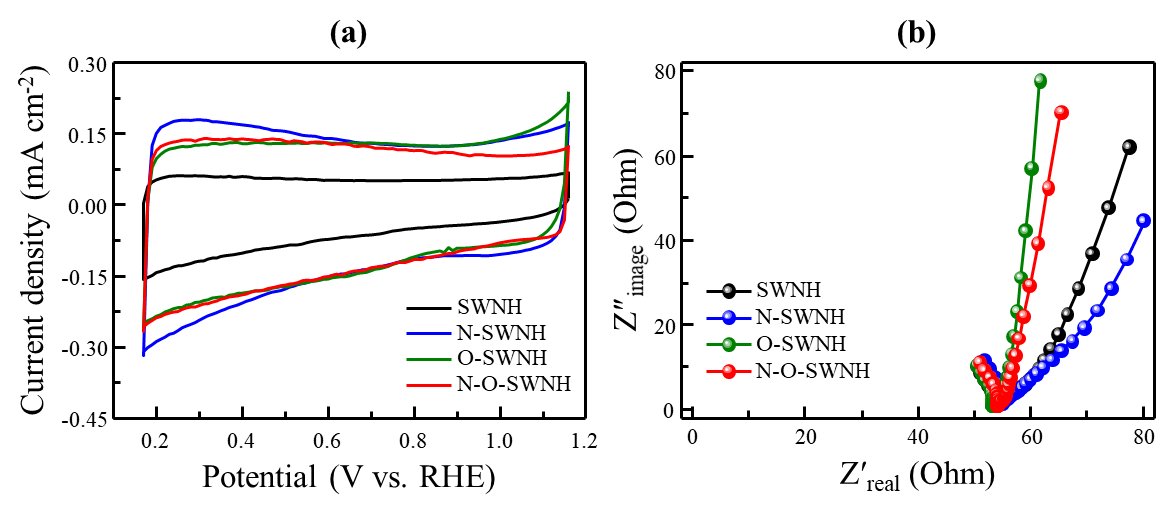


Figure S6. (a) Cyclic voltammetry at scan rate of 5 mV s-1 and (b) Nyquist plot of SWNH, O-SWNH, N-SWNH, and N-O-SWNHs in 0.1 M nitrogen bubbled KOH electrolyte


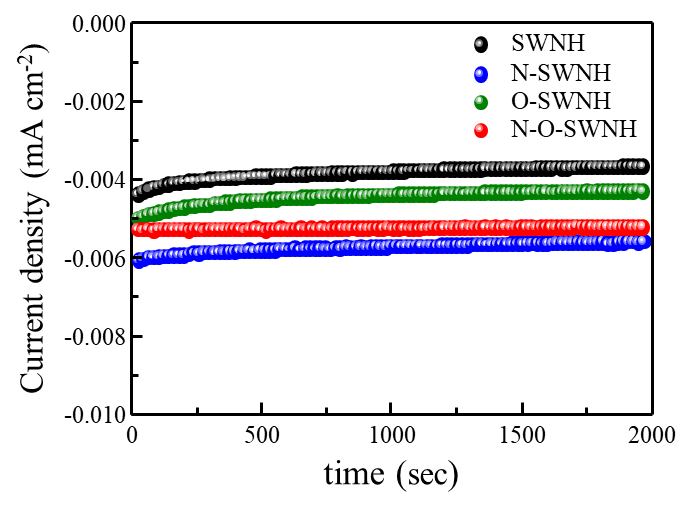


Figure S7. Durability of SWNH, O-SWNH, N-SWNH, and N-O-SWNHs in 0.1 M oxygen-saturated KOH at 0.6 V vs. RHE at 1600 rpm for 2000 sec.

Table S1. Elemental compositions and bonding configurations of pristine and N-doped SWNHs.

|  | Element composition ratio (at%) | | | | | |
| --- | --- | --- | --- | --- | --- | --- |
|  | C 1s | N 1s | | | | O 1s |
|  |  | Pyridinic-N  (398.5 eV) | Pyrrolic-N  (399.8 eV) | Quaternary-N  (401.2 eV) | Oxidized-N  (403.2 eV) |  |
| SWNH | 90.12 | ⸺ | | | | 9.88 |
| N-SWNH | 87.85 | 3.15 | 1.96 | 1.40 | 0.87 | 4.77 |
| O-SWNH | 87.80 | ⸺ | | | | 12.20 |
| N-O-SWNH | 81.92 | 7.18 | 3.03 | 2.02 | 1.03 | 4.82 |

1. *Corresponding Authors

   E-mail address: yak@chonnam.ac.kr (Y.A. Kim), cmyang1119@kist.re.kr (C.M. Yang) [↑](#footnote-ref-2)
